# Group-based trajectory modeling of platelet dynamics in sepsis: from phenotypic identification to the exploration of prognostic mediators

**DOI:** 10.3389/fmed.2026.1761545

**Published:** 2026-02-25

**Authors:** Xi Zhang, Jundan Cai, Chunhui Ni, Yingying Ma, Yun Wu, Hongfeng Yang, Fang Ye

**Affiliations:** 1 Department of Critical Care Medicine, The affiliated People's Hospital of Jiangsu University, Jiangsu Province, Zhenjiang, China; 2 Department of Critical Care Medicine, The People's Hospital of Danyang, Affiliated Danyang Hospital of Nantong University, Danyang, Jiangsu Province, China

**Keywords:** group-based trajectory modeling, phenotype, platelet, sepsis, sequential (sepsis-related) organ failure assessment (SOFA)

## Abstract

Sepsis continues to impose a substantial global burden, with persistently high rates of hospitalization and mortality. A major challenge in advancing sepsis therapeutics lies in its inherent clinical and biological heterogeneity. Recent research has increasingly shifted from static biomarker assessment toward the identification of dynamic trajectory subtypes to better capture this heterogeneity.

**Objective:**

This study aims to characterize the temporal patterns of platelet count changes during the first three days after admission among 280 sepsis patients using group-based trajectory modeling (GBTM), assess their association with clinical outcomes, and explore potential mediating pathways.

**Methods:**

We retrospectively analyzed data from 280 sepsis patients admitted to the Department of Critical Care Medicine at Jiangsu University Affiliated People's Hospital between September 2022 and December 2024. Baseline demographics, clinical characteristics, and serial platelet counts were collected. Missing data were addressed using multiple imputation techniques. GBTM was applied to identify distinct trajectory patterns of platelet counts. The association between each trajectory and 28-day all-cause mortality was evaluated using multivariable Cox proportional hazards regression models. Additionally, mediation analysis was performed to investigate potential mechanisms underlying these associations.

**Results:**

GBTM revealed three distinct platelet count trajectories: persistent low level (71.79%), high-level decline (20.00%), and rebound rise (8.21%). Patients in the persistent low-level group exhibited significantly higher 28-day all-cause mortality compared to the other two groups (71.63% vs. 55.36% vs. 13.04%, *p* < 0.05) and had the shortest median survival time. After adjusting for key confounders, Cox regression showed that, relative to the persistent low-level group, the high-level decline group had a 42% lower risk of death (*HR* = 0.58, 95% CI: 0.36–0.92, *p* = 0.02), while the rebound rise group demonstrated an 93% reduction in mortality risk (*HR* = 0.07, 95% CI: 0.02–0.26, *p* < 0.001). Mediation analysis indicated that the effect of platelet trajectories on 28-day mortality may be partially mediated through changes in Sequential Organ Failure Assessment (SOFA) score and log-transformed APTT.

**Conclusion:**

Dynamic modeling of platelet count trajectories enables effective identification of clinically meaningful subphenotypes in sepsis patients, offering a robust framework for prognosis prediction. This approach supports refined risk stratification and personalized management strategies, thereby providing novel insights into the pathophysiology and clinical care of sepsis.

## 1 Introduction

Sepsis is fundamentally characterized by a dysregulated host immune response to infection, initiated by excessive inflammatory stimulation and often progressing to organ dysfunction (1). Although the incidence and mortality of sepsis vary by geographic region and level of national development, ICU mortality remains persistently high, typically ranging from 30% to 46% (2). Accumulating evidence indicates that platelets play a pivotal role in the pathophysiology of sepsis (3). Upon activation during sepsis, platelets release procoagulant and pro-inflammatory mediators—components that contribute to host defense but, when excessively activated, may trigger disseminated intravascular coagulation (DIC), leading to organ dysfunction (4). DIC-induced organ failure primarily arises from fibrin deposition in the microvasculature and suppression of fibrinolysis, both of which impair tissue oxygenation and ultimately result in multi-organ dysfunction (5).

Platelets originate from bone marrow megakaryocytes and are non-nucleated cell fragments generated through cytoplasmic shedding. They are small in size but abundant in number, constituting the second most numerous cellular component in circulating blood after red blood cells (6). Traditionally, platelets are well - known for their hemostatic and thrombotic functions. However, their functions extend far beyond hemostasis and thrombus formation. Their crucial role in host immune regulation is attracting increasingly wide - spread research interest (7). Platelets act as a bridge between hemostasis and immunity, and their immunomodulatory functions are mainly reflected in the following two pathways: On the one hand, platelets can recognize the fibrinogen - binding proteins expressed by pathogens via their surface hemostatic receptor GPIIb/IIIa, induce platelet aggregation, and subsequently promote the occurrence of disseminated intravascular coagulation (DIC), ultimately driving the process of multiple organ failure. On the other hand, platelets also express various immune - related receptors such as FcγRIIa and Toll - like receptors (TLR), and directly participate in pathogen recognition and intracellular signal transduction, thus playing a significant role in the innate immune response and platelet activation process (8). More and more studies have revealed that a decrease in platelet count is clearly associated with a poor prognosis in patients with sepsis (9, 10). In addition, the study by Wang et al. further established a significant non - linear relationship between platelet count and 30 - day in - hospital mortality, with the optimal turning point being 176 × 10^9^/L. On both sides of this turning point, the changes in platelet count have opposite effects on the risk of death: below this value, an increase of 10 × 10^9^/L is associated with a 6% reduction in risk; above this value, the same increase is associated with a 1% increase in risk (11). This intricate nonlinear relationship indicates that distinct patients may follow markedly different dynamic evolution trajectories of platelet counts. Moreover, specific trajectory patterns could predict prognosis more precisely than the values at any single, isolated time point. Recently, a review by Aseman et al. put forward the proposal that in sepsis, platelet production, consumption, and functional status undergo rapid temporal changes. This suggests that static measurements are likely to overlook significant temporal heterogeneity and its clinical implications. Therefore, it is essential to characterize the longitudinal dynamic changes of platelets to ascertain whether distinct platelet trajectory patterns can more effectively reflect the underlying pathophysiological mechanisms, thereby overcoming the limitations of isolated measurements and enhancing prognostic stratification (12).

Group-based trajectory modeling (GBTM) is a finite mixture modeling approach that identifies latent subgroups of patients with similar longitudinal trajectories over time. Unlike dimension-reduction approaches that transform data into abstract latent variables, GBTM directly models repeated measurements on the original scale, thereby preserving clinical interpretability. This allows the identification of distinct temporal patterns, clinically meaningful subphenotypes, and dynamic heterogeneity (13). GBTM has been extensively utilized in fields such as medicine and psychology and is especially well - suited for analyzing longitudinal data (14). Sepsis is a heterogeneous syndrome characterized by extensive and multi - dimensional clinical and biological features. Additionally, the physiological response to infection is dynamic and evolves rapidly within minutes to hours (15, 16). From a systems perspective, sepsis can be conceptualized as a Complex Adaptive System characterized by nonlinear regulation, feedback interactions, and time-dependent progression across multiple biological scales. These properties generate heterogeneous clinical trajectories that cannot be adequately captured by static assessments.

Given its unique edge in capturing dynamic heterogeneity, GBTM offers a novel possibility for the in - depth analysis of the pathological process of sepsis. It can identify clinical subgroups with distinctive evolution patterns, thus revealing patient groups that demand key attention. By categorizing patients into trajectory categories corresponding to different prognostic outcomes, this approach facilitates more accurate risk stratification and provides a foundation for formulating individualized treatment strategies, particularly in heterogeneous syndromes such as sepsis where platelet behavior reflects the evolving balance between immune activation and coagulation dysregulation. Therefore, to more comprehensively explore the association between platelet dynamic evolution and prognosis in patients with sepsis, this study measured platelet counts at multiple time points and applied group-based trajectory modeling to identify sepsis subphenotypes with distinct evolution patterns, followed by comparisons of their clinical characteristics.

## 2 Materials and methods

### 2.1 Study design and participants

Patients with sepsis admitted to the Department of Intensive Care Medicine at the Affiliated People's Hospital of Jiangsu University from September 2022 to December 2024 were selected as study subjects. To construct an inception cohort, all patients were enrolled at the early stage of a newly diagnosed infection leading to sepsis, defined as within 24 h of diagnosis or ICU admission. Patients with chronic, prolonged infectious conditions or pre-existing organ failure states were excluded to ensure a relatively homogeneous baseline and consistent temporal starting point for trajectory modeling. **Inclusion Criteria**: (1) Meet the sepsis 3.0 definition and diagnostic criteria issued by the Society of Critical Care Medicine (USA) and the European Society of Intensive Care Medicine (1); (2) Age ≥ 18 years. (3) Length of hospital stay ≥ 3 days. **Exclusion Criteria**: (1) Age < 18 years; (2) Length of hospital stay < 3 days; (3) History of key diseases and treatments affecting platelet count: Patients with a history of hematological malignancies (e.g., leukemia, lymphoma, myelodysplastic syndromes); Patients with chronic liver failure (Child-Pugh class C) or a known history of cirrhosis; Patients with a history of splenectomy; Patients who received chemotherapy or radiotherapy within 30 days prior to ICU admission; Patients who received therapeutic anticoagulation (e.g., for atrial fibrillation or venous thromboembolism) or antiplatelet therapy (e.g., clopidogrel, aspirin) within 7 days prior to hospital admission. (4) Specific patient groups and treatment interferences: Patients who received blood transfusions before or after ICU admission; Patients who underwent major surgery (e.g., cardiothoracic surgery, organ transplantation) within 48 hours before or after ICU admission; Pregnant women or lactating women. (5) To construct the trajectory model, participants with fewer than 3 platelet count measurements within the first 3 days of hospital admission were excluded. This study protocol was reviewed and approved by the Ethics Committee of the Affiliated People's Hospital of Jiangsu University, and was conducted in accordance with the ethical standards of the Declaration of Helsinki. Considering its retrospective nature, the Ethics Committee granted exemption from obtaining informed consent.

### 2.2 Data collection

The data collected in this study included basic information of patients, clinical characteristics (gender, age, severity of illness score [SOFA score, APACHE-II score, Glasgow coma Scale] and comorbidities [hypertension, diabetes, heart failure]). The first laboratory test within 6 hours of ICU admission covers: Lymphocyte count, neutrophil count, monocyte count, total bilirubin (TBil), high density lipoprotein (HDL), low density lipoprotein (LDL), oxygenation index, albumin (ALB), alanine aminotransferase (ALT), aspartate aminotransferase (AST), serum creatinine (Cr), blood urea nitrogen (BUN), interleukin (IL) Serum levels of IL-6, PCT, WBC, Lac, CRP and Na, Cl, Ca, K ions were measured.

Coagulation parameters including mean platelet volume (MPV), fibrinogen (FIB), prothrombin time (PT), activated partial thromboplastin time (APTT) and D-Dimer levels were recorded within 6 hours after admission. Platelet count was dynamically monitored at the following time points: the first platelet count within 6 hours after admission (initial PLT), the baseline platelet count within 1 day before ICU admission (basal PLT), the continuous platelet count recorded within 1–3 days after admission (PLT 24h/48h/72h) and the lowest platelet count during the course of the disease (min PLT). Circadian rhythms in platelet counts and platelet activity have been reported. To minimize the potential bias, platelet samples at our institution were collected at approximately 8 am each day. Therefore, the three measurements required within the first 3 days were obtained once daily at consistent time points (17).

Prognostic indicators included: length of ICU stay, total length of hospital stay; The primary end point was 28-day all-cause mortality, with survival time recorded. Complications (renal insufficiency, shock) and dynamic recovery of platelet were recorded.

### 2.3 Study outcomes

The primary outcome measure of this study was 28-day all-cause mortality and 28-day survival time. Secondary outcome measures included ICU length of stay, total hospital length of stay, incidence of complications (such as renal dysfunction and shock), and platelet recovery status.

### 2.4 Dynamic trajectory analysis of platelets

The Group-Based Trajectory Model (GBTM) is a statistical method for longitudinal data analysis, with its core focus on identifying individual subgroups that exhibit similar change trajectories over time in repeatedly measured variables (18). In this study, R software was used to analyze the longitudinal change trajectories of platelets based on patients' initial platelet count (PLT) and continuous measurements on the 1st, 2nd, and 3rd days after admission (PLT 24h, PLT 48h, PLT 72h) using the Group-Based Trajectory Model (GBTM). This model was employed to determine the optimal number of trajectory groups and assess the model's goodness of fit. To determine the optimal number of trajectory groups, this study comprehensively considered the following statistical criteria: the Bayesian Information Criterion (BIC) and the Akaike Information Criterion (AIC), where smaller values indicate better model fit; the Average Posterior Probability (AvePP), which requires a value greater than 0.7 to ensure accurate classification of individuals into the corresponding trajectory subgroups; additionally, the sample size of each trajectory subgroup must be no less than 5% of the total sample size. Furthermore, the simplicity and clinical interpretability of the model are also important decision-making criteria.

### 2.5 Statistical analysis

Categorical variables were expressed as frequency (percentage), and intergroup comparisons were performed using chi-square test or Fisher's exact test. Continuous variables conforming to normal distribution were expressed as mean ± standard deviation, and intergroup comparisons were conducted using one-way analysis of variance (ANOVA); continuous variables with non-normal distribution were expressed as median and interquartile range (IQR), and intergroup comparisons were performed using Wilcoxon-Mann-Whitney rank sum test. Missing data were handled using multiple imputation. To evaluate the independent association between different platelet trajectories and 28-day mortality risk, this study applied the Schoenfeld residual method to assess the proportional hazards assumption, constructed a multivariate Cox proportional hazards regression model, and adjusted for potential confounding factors such as age, gender, APACHE II score, and SOFA score. Spearman rank correlation was used to assess the correlation between variables. To correct for multicollinearity, variables with a variance inflation factor greater than 7 were excluded when constructing the final model. In addition, survival analysis was used to compare survival rate differences among different subgroups. To further explore the robustness of the results, continuous variables such as age, SOFA score, GCS score, and APACHE II score were dichotomized based on the median, and subgroup analyses were conducted in combination with factors such as gender, platelet recovery status, and comorbidity status to evaluate the impact of these factors on the primary outcome. Additionally, mediating effect analysis was performed to determine whether platelets mediate the survival of septic patients through other variables. Statistical significance was set at *p* < 0.05 (two-tailed). All statistical analyses were performed using R 4.2.0 (R development Core Team, Vienna, http://www.R-project.org) and SPSS 26.0.

## 3 Result

### 3.1 Platelet trajectory features

**Figure 1:**
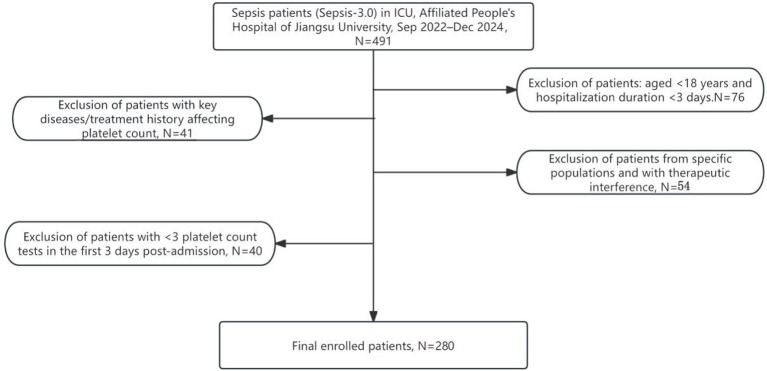
Flowchart of patient enrollment.

The study initially screened 491 sepsis patients admitted to the Department of Intensive Care Medicine of the Affiliated People's Hospital of Jiangsu University from September 2022 to December 2024. After review according to the predetermined inclusion and exclusion criteria, a total of 280 patients were finally determined to constitute the study cohort (Figure 1).

Based on platelet counts measured 6 h after patient admission and on days 1, 2, and 3, we conducted a Group-Based Trajectory Modeling (GBTM) analysis. Model fitting indicators showed that as the number of trajectory groups increased from 1 to 5, both AIC and BIC values first decreased and then increased, reaching their minimum when the number of groups was 4. Although the average posterior probability (AvePP) of all four-group models exceeded the conventional standard of 0.7, one group had a sample proportion below 5%. Considering that the model needs to balance statistical power and clinical interpretability, the optimal number of groups was finally determined to be 3. The AvePP values of the three-group models were 0.90, 0.86, and 0.83, respectively, indicating the best model fit and classification accuracy (see Supplementary Table S1).

**Figure 2:**
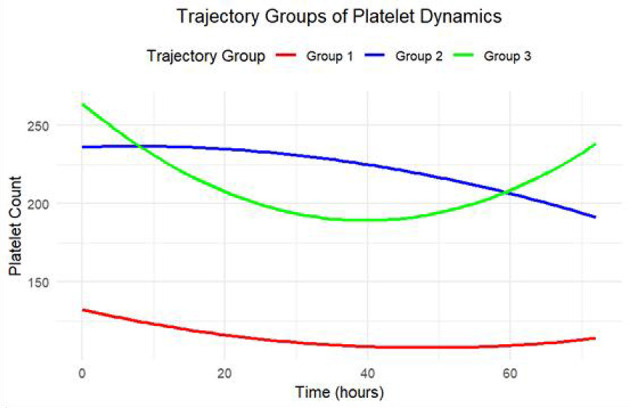
Group-based trajectory modeling of platelet counts during the first 3 days in the ICU.

According to trajectory characteristics, patients were categorized into three groups: the first group (*n* = 201, 71.79%) was defined as the ‘Persistent Low Level Group', whose platelet counts remained at a low level throughout, showing a trend of slow decline followed by recovery but with overall mild fluctuations; the second group (*n* = 56, 20.00%) was the ‘High Level Decline Group', characterized by an initially high level followed by a continuous decline; the third group (*n* = 23, 8.21%) was the ‘Rebound Increase Group', in which platelet counts experienced an initial decline followed by a rapid upward trend (Figure 2).

### 3.2 Baseline characteristics of three platelet trajectory categories

The intergroup comparison of baseline data and clinical indicators among the three patient groups showed that their general demographic characteristics and distribution of major comorbidities were balanced. The average age of the participants was 75 (65–82.25) years, with male patients accounting for 65.71% of the study population. The median age of the three groups was similar, and there were no statistically significant differences in gender composition, history of hypertension, diabetes, and heart failure (*p* > 0.05), indicating good comparability of the groups in terms of baseline clinical characteristics.

In terms of platelet function and coagulation, there were significant differences in indicators among the groups. Among them, the minimum platelet count (minPLT) of the third group (rebound increase group) was significantly higher than that of the first two groups (*p* < 0.01), while its mean platelet volume (MPV) was the smallest among the three groups. Regarding coagulation parameters, the overall median of APTT and D-dimer (D-Dimer) in the persistent low level group reached the highest among the three groups, at 43.2 s (36.5, 50.1) and 4.22 mg/L (2.44, 8.59) respectively, and the intergroup differences were statistically significant.

**Figure 3:**
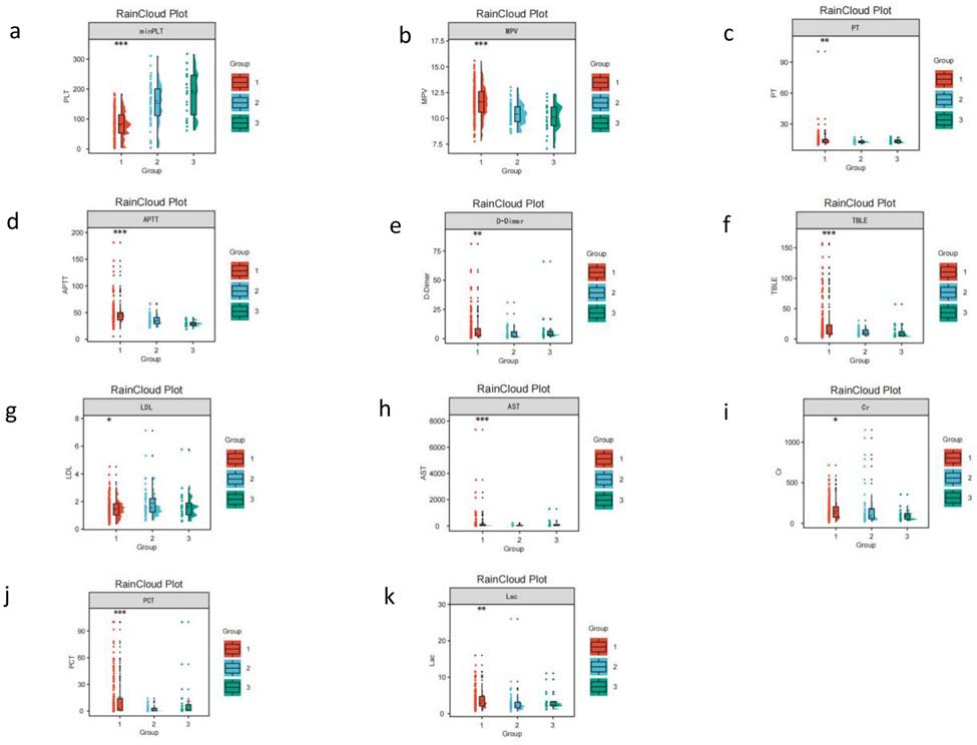
Distribution of key clinical measures across platelet trajectory subgroups (raincloud plots). **a, b**: the minimum platelet count (minPLT) of the third group (rebound increase group) was significantly higher than that of the first two groups, while its mean platelet volume (MPV) was the smallest among the three groups; **c**: PT showed significant differences among the groups; **d,e**: the overall median of APTT and Ddimer (D-Dimer) in the persistent low level group reached the highest among the three groups, at 43.2s and 4.22 mg/L; **f, g, h, i**: total bilirubin (TBil), low-density lipoprotein (LDL), aspartate aminotransferase (AST), and creatinine (Cr) all showed significant differences among the groups; **j, k**: procalcitonin (PCT) was highest in the first group, and the lactate (Lac) level of patients in this group was also high.

In terms of organ function and inflammatory indicators, intergroup differences were mainly manifested in SOFA score and multiple biochemical indicators. The SOFA score of the second group (high level decline group) was the lowest among the three groups, while that of the first group (persistent low level group) was the highest. In addition, total bilirubin (TBil), aspartate aminotransferase (AST), creatinine (Cr), and low-density lipoprotein (LDL) all showed significant differences among the groups. Regarding inflammatory indicators, procalcitonin (PCT) was highest in the first group (2.85 (0.82–14.07) mg/L), and the lactate (Lac) level of patients in this group was also high. Their intergroup trend was: persistent low level group > rebound increase group > high level decline group (Table 1, Figure 3, complete with Supplementary Table S2).

**Table 1:** Comparative analysis of baseline and clinical features across platelet trajectory subgroups.

		**Overall**	**Group1**	**Group2**	**Group3**	***p***
Sample Size	–	280	201	56	23	–
**Baseline characteristics**						
Age (years)	–	75 (65-82.25)	76 (65–83)	73.5 (60.5–80)	73.52 ± 10.04	0.28
GCS (points)	–	12 (5–15)	12 (5–15)	13.5 (5.75–15)	12 (6–15)	0.82
SOFA (points)	–	8 (5–12)	9 (6–13)	6 (4–9)	8 (4.5–12)	< 0.01
APACHE II (points)	–	20 (16–27)	20 (16–27)	21.5 (14.75–28.25)	20.43 ± 7.17	0.56
Gender (%)	Female	96 (34.29)	69 (34.33)	16 (28.57)	11 (47.83)	0.26
	Male	184 (65.71)	132 (65.67)	40 (71.43)	12 (52.17)	–
Hypertension (%)	Yes	142 (50.71)	100 (49.75)	31 (55.36)	11 (47.83)	0.73
	No	138 (49.29)	101 (50.25)	25 (44.64)	12 (52.17)	–
Diabetes (%)	Yes	89 (31.79)	64 (31.84)	18 (32.14)	7 (30.43)	0.99
	No	191 (68.21)	137 (68.16)	38 (67.86)	16 (69.57)	–
Cardiac insufficiency (%)	Yes	77 (27.50)	56 (27.86)	14 (25.00)	7 (30.43)	0.87
	No	203 (72.50)	145 (72.14)	42 (75.00)	16 (69.57)	–
**Serum indicators**						
Neutrophil (10^9^/L)	–	10.28 (7.02–15.63)	9.7 (6.8–15.7)	11.58 ± 6.57	12.8 (10.49–16.55)	0.21
Lymphocyte (10^9^/L)	–	0.64 (0.4–0.95)	0.63 (0.4–0.9)	0.6 (0.5–0.95)	0.94 ± 0.6	0.18
Monocyte (10^9^/L)	–	0.5 (0.28–0.75)	0.4 (0.21–0.72)	0.5 (0.35–0.77)	0.5 (0.38–1)	0.19
PLT (10^9^/L)	–	155.5 (112.75–214.25)	132.22 ± 49.07	248.25 ± 60.16	286.52 ± 68.27	< 0.01
Base PLT (10^9^/L)	–	192 (142–245)	166.17 ± 52.7	261.46 ± 70.02	316.87 ± 51.96	< 0.01
24h PLT (10^9^/L)	–	134.5 (96–193)	114.61 ± 43.43	250.52 ± 46.13	211.3 ± 71.5	< 0.01
48h PLT (10^9^/L)	–	126 (88.75–178.25)	106.93 ± 46.28	229.32 ± 66.72	212.26 ± 97.65	< 0.01
72h PLT (10^9^/L)	–	133.5 (83.75–192.75)	114.75 ± 54.6	199.2 ± 82.69	260.74 ± 95.62	< 0.01
Min PLT (10^9^/L)	–	92.5 (60.75–141)	83.46 ± 43.36	153.46 ± 73.32	186 ± 76.7	< 0.01
MPV (fL)	–	11.24 ± 1.55	11.6 ± 1.54	10.42 ± 1.04	10.11 ± 1.35	< 0.01
PT (s)	–	13.25 (12.1–14.8)	13.5 (12.2–15.1)	12.79 ± 1.36	13.5 ± 2.07	< 0.01
APTT (s)	–	40.5 (32.2–46.32)	43.2 (36.5–50.1)	32.85 (29.05–40.52)	29.65 ± 5.05	< 0.01
FIB (g/L)	–	4.44 (3.55–5.6)	4.44 (3.68–5.69)	4.6 ± 1.69	4.1 ± 1.73	0.13
D-dimer (μg/ml)	–	3.58 (2.18–8.28)	4.22 (2.44–8.59)	2.41 (1.52–6.03)	3.2 (2.63–6.23)	0.01
**Organ function-related indicators**						
ALB (g/L)	–	29.5 (26.2–32.92)	29.7 ± 5.56	29.6 (27.3–33.73)	27.75 (25.23–29.6)	0.10
ALT (U/L)	–	25 (14–53.08)	26 (15–55)	20 (12–36.25)	36 (12.5–62)	0.10
AST (U/L)	–	37 (23–75.75)	40 (25–92)	26 (18.75–43.25)	43 (23.75–88)	< 0.01
Cr (μmol/L)	–	112.5 (67.75–185)	124 (74.51–205.2)	89 (54.75–177.36)	86 (51.35–121.5)	0.01
BUN (mmol/L)		10.44 (7–16.98)	11 (7.3–17.51)	8.85 (6.4–15.03)	11.26 ± 7.84	0.12
**Inflammatory markers**						
IL-6 (pg/ml)	–	115.54 (22.15–264.82)	119.69 (25.51–262.11)	104.61 (18.52–302.58)	87.14 (11.37–235.2)	0.62
PCT (mg/L)	–	2.23 (0.47–8.7)	2.85 (0.82–14.07)	1.06 (0.21–3.27)	2.06 (0.42–7.32)	< 0.01
WBC (10^9^/L)		11.3 (7.96–17.2)	11.3 (7.98–17.2)	12.59 ± 6.6	15.23 ± 8.05	0.27
CRP (mg/L)	–	90.66 (30.12–162.09)	91.34 (33.9–167.2)	69.31 (20.81–127.58)	108.93 (82.89–163.58)	0.08
Lac (mmol/L)	–	2.8 (2–4.2)	3 (2.1–4.7)	2.2 (1.6–3.12)	2.5 (2.15–3.25)	< 0.01

Group 1: Persistent Low Level Group; Group 2: High Level Decline Group; Group 3: Rebound Increase Group; p < 0.05 was considered statistically significant.

### 3.3 Comparison of characteristics among patients with different outcomes

**Figure 4:**
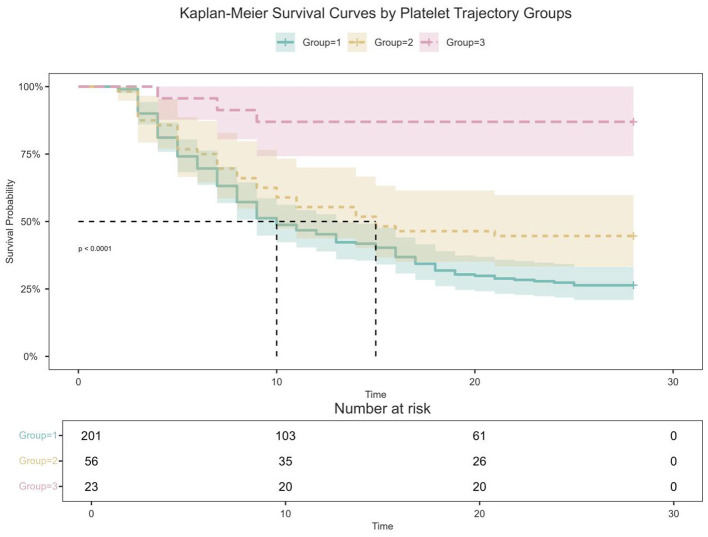
Kaplan-Meier survival cerves by platelet trajectory groups.

Based on the survival status of patients 28 days after hospital admission, this study divided them into a survival group (*n* = 98, 35%) and a non-survival group (*n* = 182, 65%). Intergroup comparisons showed that, compared with the non-survival group, patients in the survival group had relatively milder organ dysfunction and inflammatory responses, specifically manifested as significantly lower levels of SOFA scores, total bilirubin (TBil), aspartate aminotransferase (AST), creatinine (Cr), blood urea nitrogen (BUN), procalcitonin (PCT), and lactate (Lac). Regarding coagulation and platelet indices, the survival group had lower levels of activated partial thromboplastin time (APTT), mean platelet volume (MPV), fibrinogen (FIB), and D-dimer (D-Dimer), while platelet counts (including initial PLT, baseline basePLT, and minimum minPLT during the course of illness) and serum potassium ion levels were higher (Figure 4). In addition, the ICU length of stay and total hospital length of stay in the survival group were significantly shorter than those in the non-survival group, and platelet recovery was also Obviously better (*p* < 0.05 for all comparisons). Unexpectedly, there was no statistically significant difference in the incidence of complications such as renal insufficiency and shock between the two groups (*p* > 0.05) (Table 2, complete with Supplementary Table S3).

**Table 2:** Baseline characteristics of patients by 28-day survival status.

		**Survival Group**	**Non-Survival Group**	***p***
Sample Size	–	98	182	–
**Baseline characteristics**				
Age (year)	–	75.5 (67.25–82)	75 (63–83)	0.63
GCS (points)	–	12 (6–15)	12 (5–15)	0.99
SOFA (points)	–	7 (4–11)	9 (6–13)	< 0.01
APACHE II (points)	–	20 (15.25–26.75)	21 (16–27)	0.24
Gender (%)	Female	31 (31.63)	65 (35.71)	0.58
	Male	67 (68.37)	117 (64.29)	–
Hypertension (%)	Yes	53 (54.08)	89 (48.90)	–
	No	45 (45.92)	93 (51.10)	0.48
Diabetes (%)	Yes	29 (29.59)	60 (32.97)	–
	No	69 (70.41)	122 (67.03)	0.66
Cardiac insufficiency (%)	Yes	30 (30.61)	47 (25.82)	–
	No	68 (69.39)	135 (74.18)	0.47
**Serum indicators**				
Neutrophil (10^9^/L)	–	9.73 (6.45–14.65)	10.55 (7.35–16.67)	0.22
Lymphocyte (10^9^/L)	–	0.7 (0.5–1.15)	0.6 (0.38–0.9)	0.02
Monocyte (10^9^/L)	–	0.5 (0.32–0.8)	0.4 (0.2–0.72)	0.05
PLT (10^9^/L)	–	188 (147.5–256)	134 (98.25–189.75)	< 0.01
basePLT (10^9^/L)	–	212 (174–286.25)	170.5 (129.25–210)	< 0.01
24hPLT (10^9^/L)	–	160.5 (131–236)	116 (84.5–171)	< 0.01
48hPLT (10^9^/L)	–	148.5 (110.25–207.75)	106.5 (78.25–160.5)	< 0.01
72hPLT (10^9^/L)	–	160 (115.75–209)	112.5 (70–181)	< 0.01
minPLT (10^9^/L)	–	121 (83.25–157.75)	82.5 (52–128.25)	< 0.01
MPV (fL)	–	10.83 ± 1.46	11.46 ± 1.55	< 0.01
PT (s)	–	13.32 ± 1.94	13.4 (12.2–15.07)	0.08
APTT (s)	–	33.6 (28.15–42.42)	43 (36.6–48.7)	< 0.01
FIB (g/L)	–	3.72 ± 1.06	5 (4.03–6.23)	< 0.01
D-Dimer (μg/ml)	–	3.08 (1.8–6.79)	4.04 (2.41–9.12)	0.02
**Organ function-related indicators**				
TBil (μmol/L)	–	10 (7.32–16.32)	12.6 (8.4–21.27)	0.01
HDL (mmol/L)	–	1.11 ± 0.45	1.04 ± 0.42	0.19
LDL (mmol/L)	–	1.43 (1.09–1.74)	1.48 (1.05–1.91)	0.51
Oxygenationindex (mmHg)	–	249.13 ± 107.25	234.75 (169.44–311.88)	0.97
ALB (g/L)	–	28.9 (26–32.2)	29.9 (26.52–33.32)	0.17
ALT (U/L)	–	21 (12–45.75)	27.5 (15–54)	0.15
AST (U/L)	–	33.75 (22–52.75)	39 (23.7–96.5)	0.04
Cr (μmol/L)	–	88.9 (55.25–135.75)	131.01 (75.5–216.38)	< 0.01
BUN (mmol/L)	–	8.9 (6.4–14.5)	11.5 (7.55–17.28)	0.02
**Inflammatory markers**				
IL-6 (pg/mL)	–	68.81 (15.65–340.96)	135.54 (31.56–241.09)	0.19
PCT (mg/L)	–	1.62 (0.28–5.86)	2.83 (0.85–11.93)	< 0.01
WBC (10^9^/L)	–	11.3 (7.25–16.38)	11.3 (8.31–17.63)	0.29
CRP (mg/L)	–	92.03 (23.34–146.24)	90.42 (37.31–167.43)	0.44
Lac (mmol/L)	–	2.6 (1.9–3.27)	3 (2.02–4.8)	0.01
Na (mmol/L)	–	139.13 (134.9–142.45)	139.94 (135.9–142.48)	0.24
CI (mmol/L)	–	101.32 ± 7.97	101.05 (98.12–105.71)	0.76
Ca (mmol/L)	–	1.94 (1.83–2.09)	1.96 (1.85–2.1)	0.72
K (mmol/L)	–	4.1 (3.7–4.52)	3.89 ± 0.64	< 0.01
**Outcome-related indicators**				
ICU length of stay (days)	–	18 (9–30)	7 (4–11)	< 0.01
Hospital length of stay (days)	–	26 (16-39)	10.5 (7-19)	< 0.01
28-day survival time (days)	–	28 (28–28)	7 (4–12)	< 0.01
Platelet recovery (%)	Yes	88 (89.80)	124 (68.13)	< 0.01
	No	10 (10.20)	58 (31.87)	–
AKI (%)	Yes	32 (32.65)	78 (42.86)	0.12
	No	66 (67.35)	104 (57.14)	–
Shock (%)	Yes	33 (33.67)	68 (37.36)	0.63
	No	65 (66.33)	114 (62.64)	–

P < 0.05 was considered statistically significant.

### 3.4 Association between different trajectory groups and clinical outcomes

The results showed that the 28-day all-cause mortality rate of the ‘Persistent Low Level Group' (Group 1) was significantly higher than that of the other two groups, reaching as high as 71.63%, with the shortest median survival time (*p* < 0.05), followed by the ‘High Level Decline Group' and the ‘Rebound Increase Group'. The ICU lengths of stay for the three groups were 8 (5–16), 10.5 (5–20.25), and 8 (5.5–15.5) days, respectively, and the total hospital lengths of stay were 14 (8–25), 16 (9.75–24.5), and 17 (13–38) days, respectively, with no significant differences among them. However, there were no statistically significant differences among the three groups in terms of the incidence of renal insufficiency, shock, and platelet recovery (Table 3).

**Table 3:** Clinical outcomes and complications by trajectory group.

		**Overall**	**Group1**	**Group2**	**Group3**	***p***
ICU Length of stay (days)	–	9 (5–17)	8 (5–16)	10.5 (5–20.25)	8 (5.5–15.5)	0.22
Hospital length of stay (days)	–	15.5 (8–25.25)	14 (8–25)	16 (9.75–24.5)	17 (13–38)	0.13
28-day survival status (%)	Death	182 (65.00)	148 (73.63)	31 (55.36)	3 (13.04)	< 0.01
	Survival	98 (35.00)	53 (26.37)	25 (44.64)	20 (86.96)	
28-day survival time (days)	–	13 (6–28)	10 (5–28)	15 (6.75–28)	28 (28–28)	< 0.01
AKI (%)	Yes	110 (39.29)	80 (39.80)	21 (37.50)	9 (39.13)	0.95
	No	170 (60.71)	121 (60.20)	35 (62.50)	14 (60.87)	–
Shock (%)	Yes	101 (36.07)	79 (39.30)	16 (28.57)	6 (26.09)	0.19
	No	179 (63.93)	122 (60.70)	40 (71.43)	17 (73.91)	–
platelet recovery (%)	Yes	212 (75.71)	149 (74.13)	43 (76.79)	20 (86.96)	0.39
	No	68 (24.29)	52 (25.87)	13 (23.21)	3 (13.04)	–

Group 1: Persistent Low Level Group; Group 2: High Level Decline Group; Group 3: Rebound Increase Group; p < 0.05 was considered statistically significant.

The Kaplan-Meier survival curves of the three groups of patients were shown in Figure 4. The 28-day follow-up results showed that different platelet trajectories were significant influencing factors for patient survival prognosis (*p* < 0.05). Among them, the persistent low level trajectory was associated with the worst survival rate, indicating that it was an important negative prognostic indicator; conversely, the rebound increase trajectory predicted the best survival outcome.

### 3.5 Multivariate cox proportional hazards regression analysis of different trajectory groups

**Figure 5:**
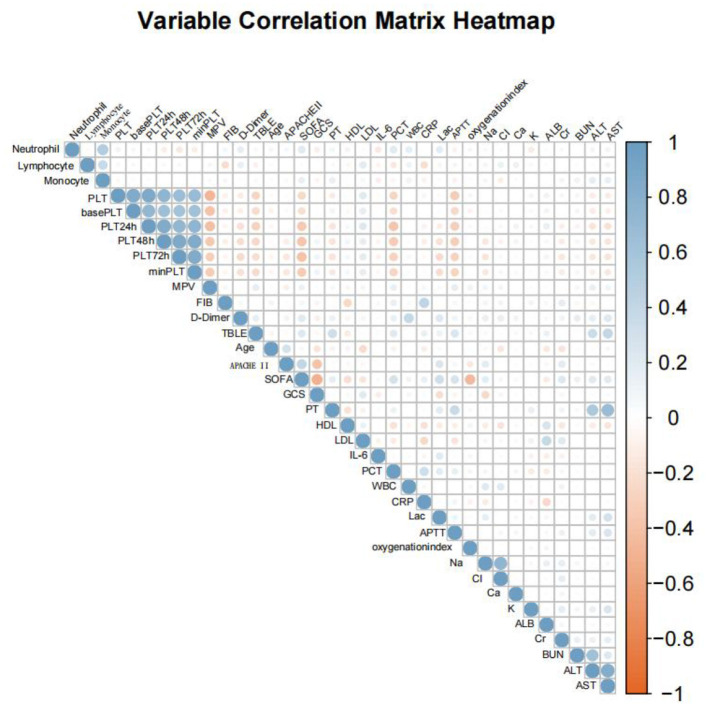
Correlation heatmap of the analyzed variables.

**Figure 6:**
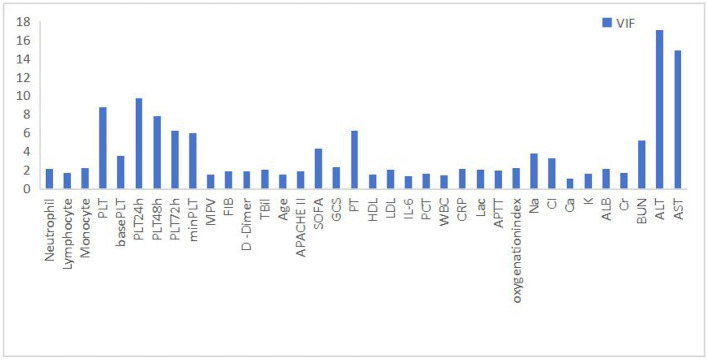
Histogram of variance inflation factor values for the analyzed variables.

In this study, Spearman's rank correlation analysis was employed to assess the associations between variables (Figure 5). To ascertain whether the platelet trajectory groups served as independent predictors of patient death, a multivariate Cox proportional hazards regression model was constructed. When incorporating variables, the variance inflation factors (VIF) for all candidate independent variables were initially computed (Figure 6). Variables with a VIF exceeding 5.0 were regarded as having severe collinearity and were excluded. Based on this, literature evidence and clinical expertise were further integrated to select the final covariates to be included in the model from the remaining variables, thereby considering both statistical robustness and clinical interpretability.

We employed a multivariate Cox proportional hazards regression model to assess the independent associations between diverse platelet trajectories and 28 - day all - cause mortality. The construction of the model adhered to a stratified adjustment strategy: The unadjusted model accounted for no confounding factors. The outcomes indicated that, using the “persistently low level group” as the reference, the mortality risks in the second and third groups were notably diminished, with hazard ratios (HRs) (95% confidence intervals [CIs]) of 0.66 (0.45–0.97) and 0.11 (0.04–0.35) respectively. Model 1 made further adjustments for age, gender, and pre - existing comorbidities; Model 2 incorporated indicators of inflammation, metabolism, and organ function (including procalcitonin [PCT], white blood cell count [WBC], C - reactive protein [CRP], lactate [Lac], oxygenation index, albumin, and creatinine) on the basis of Model 1. Eventually, considering clinical significance and statistical power, we developed Model 3 as the final refined model. Within the same clinical dimension, we prioritized the selection of indicators with stable measurements and extensive clinical utilization. The final covariates encompassed gender, age, diabetes, hypertension, cardiac insufficiency, Acute Physiology and Chronic Health Evaluation II (APACHE II) score, Glasgow Coma Scale (GCS) score, lactate, oxygenation index, serum creatinine, albumin, sodium ions, chloride ions, white blood cell count, neutrophil count, lymphocyte count, monocyte count, CRP, PCT, minimum platelet count (minPLT), mean platelet volume (MPV), fibrinogen (FIB), and D - dimer. After comprehensively adjusting for the aforementioned confounding factors, the multivariate Cox regression analysis showed that the three platelet trajectories were independently associated with 28-day all-cause mortality in patients with sepsis. Compared with the “persistently low level group”, the death risks in the “high level decreasing group” (*HR* = 0.58, 95%CI 0.36 - 0.92, *p* = 0.02) and the “rebound increasing group” (*HR* = 0.07, 95%CI 0.02 - 0.26, *p* < 0.001) were significantly reduced, with the greatest reduction in the “rebound increasing group” (Table 4).

**Table 4:** Multivariable cox regression analysis of the association between platelet trajectory groups and 28-day all-cause mortality.

	**Crude model**		**Model 1**		**Model 2**		**Model 3**	
	**HR (95%CI)**	***p***	**HR (95%CI)**	***p***	**HR (95%CI)**	***p***	**HR (95%CI)**	***p***
Group 1	1	Ref	1	Ref	1	Ref	1	Ref
Group 2	0.66 (0.45–0.97)	0.040	0.65(0.44–0.96)	0.030	0.63(0.41–0.95)	0.028	0.58(0.36–0.92)	0.020
Group 3	0.11(0.04–0.35)	< 0.001	0.11(0.03–0.34)	< 0.001	0.10(0.03–0.33)	< 0.001	0.07(0.02–0.26)	< 0.001

**Crude model**, unadjusted

**Model 1**, adjusted for age, sex, diabetes, hypertension, and cardiac insufficiency.

**Model 2**, additionally adjusted for PCT, WBC, CRP, lactate, oxygenation index, albumin, and creatinine on the basis of Model 1.

**Model 3**, further adjusted for APACHE II, GCS, lymphocyte count, neutrophil count, monocyte count, minPLT, MPV, D-dimer, fibrinogen, sodium ion, and chloride ion on the basis of Model 2.

### 3.6 Subgroup analysis of 28-day survival rate in different trajectory groups

**Figure 7:**
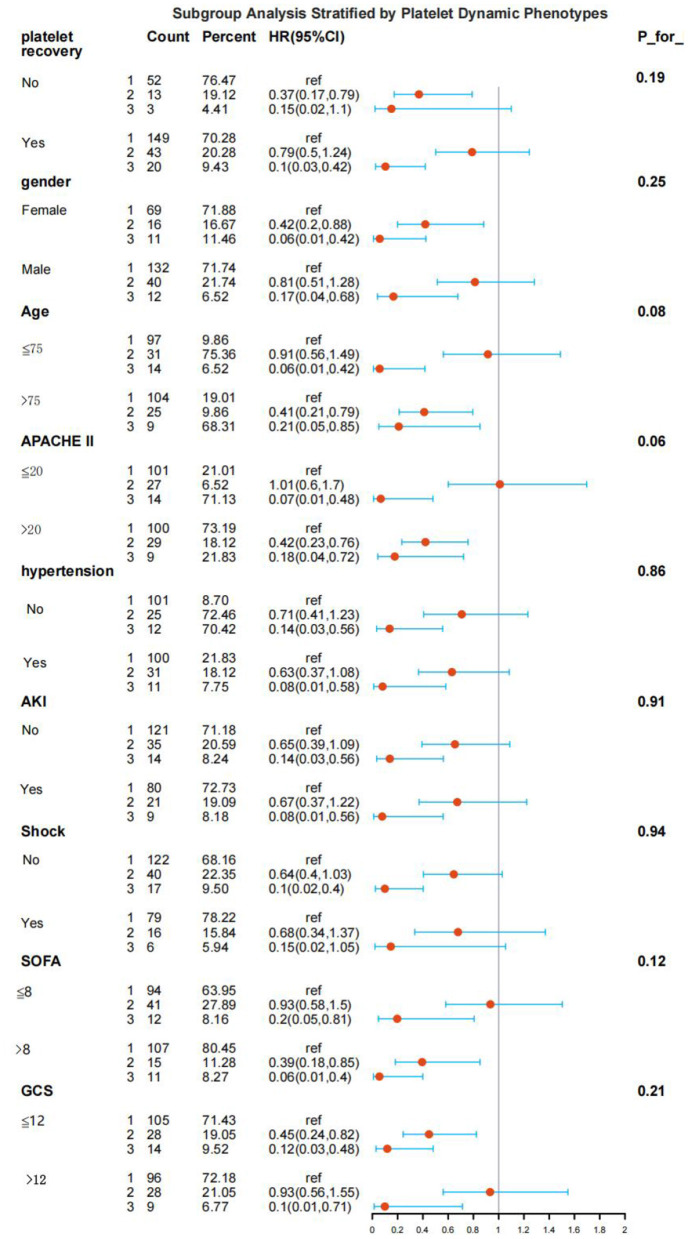
Subgroup analysis of platelet trajectories and mortality (forest plot).

To validate the robustness of the association between platelet trajectory groups and 28 - day all - cause mortality, this study carried out a series of subgroup analyses (Figure 7). The entire population was partitioned into distinct subgroups according to the medians of age, Glasgow Coma Scale (GCS), SOFA score, and Acute Physiology and Chronic Health Evaluation II (APACHE II) score, and was stratified based on gender, underlying diseases, and complications. In certain subgroups (such as specific strata with comorbid diabetes or cardiac insufficiency), owing to the small sample size, the Cox regression model failed to converge, and the confidence interval of the hazard ratio tended towards infinity. Consequently, these subgroups were excluded from the final analysis. In all the remaining evaluable subgroups, the Cox proportional hazards model was employed for analysis. The findings revealed that the direction of the association between platelet trajectory groups and 28 - day mortality remained consistent in several crucial subgroups, including patients aged over 75 years, females, those with an APACHE II score exceeding 20, a SOFA score greater than 8, and a GCS score less than or equal to 12. In other subgroups (such as those aged 75 years or younger, males, etc.), even though the differences in the hazard ratios between some groups and the reference group did not attain statistical significance, the changing trends were in line with the results of the main analysis, which further corroborated the robustness of the association between platelet trajectory and prognosis. In addition, to evaluate whether categorization or model specification might obscure potential nonlinear or dynamic effects, we conducted sensitivity analyses using restricted cubic splines (RCS). These analyses did not identify substantial nonlinear patterns, and the results remained consistent with those of the primary models (see Supplementary Figure S1, Supplementary Table S4). Taken together, the consistency across both population subgroups and alternative modeling strategies strengthens the stability and reliability of our findings. The results of the interaction test demonstrated that there were no significant interaction effects between all the included subgroup variables (including age, gender, APACHE II score, etc.) and platelet trajectory groups (*p* for interaction > 0.05). This indicated that the association between platelet trajectories and mortality remained consistent across subgroups of populations with different characteristics, and the magnitude of the effect did not vary significantly due to the differences in these baseline characteristics.

### 3.7 Exploration of the mediating effect of initial platelet levels on the risk of death

**Figure 8:**
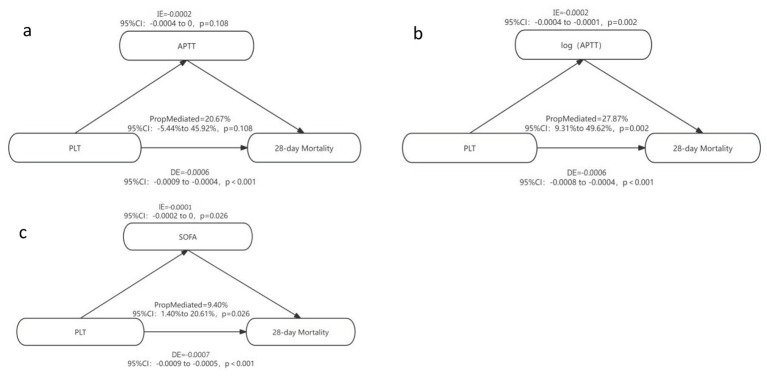
Mediating effect analysis of factors on 28-day mortality. Platelets may impact the 28 - day death risk of patients with sepsis via the path of the SOFA score and log (APTT). **a**: Log-transformed APTT exhibited a significant mediating effect on platelet-mediated mortality risk; **b**: When raw APTT was used as the mediating variable, the mediating effect was not statistically significant; **c**: The SOFA score mediated 9.4% of the association between platelets and 28-day mortality.

We employed mediation effect analysis to further investigate the mechanism through which platelet levels influence the 28 - day mortality rate of patients with sepsis. The results were presented in Figure 8. Initially, we evaluated the mediating role of activated partial thromboplastin time (APTT) in the association between platelets and 28 - day mortality. Considering that the distribution of APTT might be right - skewed, and the effect of coagulation time parameters might better conform to a multiplicative relationship rather than a linear one, we also constructed a model with logarithmically transformed activated partial thromboplastin time (log (APTT)) as the mediating variable to comprehensively evaluate its potential mediating mechanism. The analysis indicated that platelets may impact the 28 - day death risk of patients with sepsis via the path of the SOFA score and log (APTT). Specifically, the SOFA score mediated 9.4% of the association between platelets and 28 - day mortality (indirect effect *IE* = - 0.0001, 95% confidence interval *CI* = - 0.0002–0, *p* = 0.026, as depicted in Figure 8c). When the original APTT was utilized as the mediating variable, its mediating effect did not attain statistical significance (proportion of mediating effect PropMediated = 20.67%, 95%*CI* = - 5.44%−45.92%, *p* = 0.108, Figure 8a). In contrast, log (APTT) demonstrated a significant mediating effect in the platelet - mediated death risk (*IE* = - 0.0002, 95%*CI* = - 0.0004— 0.0001, *p* = 0.002, Figure 8b).

## 4 Discussion

Due to its high incidence and high mortality rate, sepsis has emerged as a major global public health challenge. The intrinsic high heterogeneity of sepsis is the primary reason why it is difficult to effectively manage this disease. Meanwhile, this highly complex heterogeneity impels us to categorize patients into more homogeneous groups sharing common biological characteristics. By doing so, it not only facilitates the formulation of individualized treatment strategies but also enables the accurate identification of high - risk patient groups (19, 20).

Although it was previously widely assumed that platelet consumption and activation were associated phenomena of sepsis, a growing body of evidence indicates that platelets play a pivotal role in the occurrence and development of sepsis and are also a core factor in sepsis - induced organ dysfunction (5). A substantial number of studies have verified that the severity of thrombocytopenia is an independent predictor of the prognosis of sepsis patients. Given that platelet detection is straightforward and the results can be obtained rapidly, it has become a highly regarded biomarker in this research field (21–23). For example, Hua et al. evaluated admission platelet levels using conventional logistic regression, while Wang et al. applied nonlinear and threshold-based models to explore associations between initial platelet levels and mortality. Although these approaches improved risk stratification, they remained inherently cross-sectional and were unable to capture the dynamic evolution of platelet changes during disease progression, as single time-point measurements cannot adequately reflect temporal variability (9, 11). Currently, there was still a dearth of in - depth research on the influence of the continuous change pattern of platelets after sepsis patients are admitted to the hospital on clinical outcomes. Therefore, this study aimed to utilize the group trajectory model to perform dynamic typing of platelets and explore its potential mediating factors.

In this study, the Group-Based Trajectory Model (GBTM) was utilized. By analyzing the platelet counts of patients within the first three days after their admission to the Intensive Care Unit (ICU), three distinct dynamic evolution patterns of platelets were successfully identified. After comprehensive analysis, the optimal model was determined to consist of three groups of trajectories: The first group was the “persistent low - level type” (*n* = 201, accounting for 71.79%). Its characteristic feature was that the platelet count remained at a relatively low level. Although it decreased gradually and then rebounded, the overall trajectory was relatively smooth with minimal fluctuations. The second group was the “high - level decline type” (*n* = 56, accounting for 20.00%). Initially, the platelet level was high, and subsequently, it exhibited a continuous downward trend. The third group was the “rebound and rise type” (*n* = 23, accounting for 8.21%). It was characterized by a rapid rebound of the platelet count following an initial decline. One of the significant contributions of this study was that it captured the dynamic evolution of platelets. In comparison to a single static measurement, the dynamic assessment of platelets might offer more advantages. Continuous dynamic assessment of platelets could more sensitively identify high - risk groups that were easily overlooked by traditional indicators, thereby providing a new theoretical basis for early and active clinical interventions targeting this group. After fully adjusting for potential confounding factors, the multivariate Cox regression analysis revealed that, when compared with the “persistent low - level group” which had the highest mortality rate, the 28 - day death risks of the “high - level decline group” and the “rebound and rise group” were significantly reduced by 42% and 93% respectively. This result strongly suggested that, apart from the static platelet count, the dynamic change trend of platelets themselves was an independent factor influencing the prognosis of sepsis. The findings of this study were in line with previous literature reports. Although there were disparities in the optimal number of groups determined according to different cohorts and models, multiple studies had arrived at a common conclusion: patients with an upward trend in platelet count had a higher survival rate, while those with a dynamic trajectory of persistently low - level platelets faced a significantly higher risk of death. This discovery further validated the crucial position of this phenotype in risk stratification (24–26).

Analysis of the clinical characteristics across the three groups revealed that patients in the persistent low-level group exhibited significantly higher SOFA scores, along with the highest levels of total bilirubin, creatinine, and blood lactate, indicating a more severe clinical condition characterized by pronounced organ dysfunction and microcirculatory impairment. In our 28-day follow-up analysis, distinct platelet trajectory phenotypes were significantly associated with survival outcomes (*p* < 0.01), whereas baseline WBC counts showed no significant variation across these phenotype groups (*p* = 0.27). Moreover, leukocyte-related variables, including differential counts and inflammatory markers, were simultaneously adjusted for in the fully multivariable model, and platelet trajectories remained independently associated with 28-day mortality. These findings suggest that platelet dynamics capture prognostic information complementary to leukocyte-based inflammatory indices rather than merely reflecting concurrent leukocyte states. WBC counts primarily reflect the instantaneous intensity of acute inflammatory responses and are highly susceptible to short-term fluctuations. In contrast, longitudinal changes in platelet counts integrate multiple processes involved in sepsis—including endothelial injury, coagulation activation, and impaired hematopoiesis— and therefore better reflect the cumulative physiological burden of disease over time. Consistent with this interpretation, the strong association between platelet trajectory dynamics and organ dysfunction observed in our study aligns closely with established pathological mechanisms reported in prior research. Vardon-Bounes et al. demonstrated that in murine models of sepsis, platelets are rapidly activated and accumulate within organ microvessels, forming platelet-rich microthrombi that lead to microvascular occlusion and tissue ischemia. Furthermore, activated platelets release substantial quantities of inflammatory mediators and procoagulant factors, amplifying systemic inflammation and coagulopathy, thereby contributing to multi-organ failure (27). Notably, the interaction between platelets and neutrophil extracellular traps (NETs) represents a pivotal mechanism exacerbating organ injury and microcirculatory dysfunction. Studies by McDonald and colleagues have shown that NETs are extensively formed in the microvasculature during sepsis, where they effectively activate thrombin and promote platelet aggregation, accelerating the development of platelet-rich microthrombi and resulting in impaired microcirculatory flow and inadequate organ perfusion. Platelets not only form direct aggregates with neutrophils but also bind to the DNA scaffold and protein components of NETs, further propagating thrombus formation. Moreover, NET-derived molecules such as histone H4 and polyphosphate act synergistically to enhance thrombin generation and platelet activation, establishing a self-amplifying “inflammation-thrombosis” loop that drives widespread microvascular dysfunction and end-organ damage. Importantly, experimental inhibition of NET formation or disruption of their structural integrity has been shown to markedly attenuate disseminated intravascular coagulation, restore microcirculatory perfusion, and mitigate organ dysfunction (28).

Although there were alternating changes in some coagulation indicators between the high-level decline group and the rebound increase group, the continuous low-level group showed the most significant abnormalities in multiple key indicators. The minimum platelet count (minPLT), activated partial thromboplastin time (APTT), and D-dimer level of this group were 83.46 ± 43.36 × 10^9^/L, 43.2 (36.5–50.1) s, and 4.22 (2.44–8.59) μg/ml, respectively, which were the highest among the three groups. This suggested the most severe coagulation dysfunction. At the same time, the procalcitonin (PCT) level, which reflected the severity of systemic inflammation and infection, also showed a similar inter-group distribution trend, further supporting that the patients in this group were in a more severe pathological physiological state. Previous study had shown that the duration of thrombocytopenia is closely related to poor prognosis (29). This finding was highly consistent with the dynamic trajectory of platelets in the continuous low-level group. This phenotype essentially reflected the imbalance in immune thrombosis regulation, characterized by excessive activation of the coagulation system (such as the significantly elevated D-dimer and prolonged APTT in this group) and uncontrolled inflammatory response (such as the significantly increased PCT) (8). These pathological processes drove microcirculation failure and multi-organ damage through mechanisms such as enhanced platelet-neutrophil interaction and extensive microthrombosis, thereby explaining the poorest clinical prognosis of this group of patients.

In the cohort comparison of patients with different prognosis, this study found that many key clinical indicators were significantly increased in patients in the non-survival group. Specifically, the coagulation function parameters (MPV, APTT, PT, FIB, D-dimer), infection and inflammation markers (procalcitonin), and tissue perfusion indexes (lactic acid) were all at high levels, and their platelet counts were difficult to return to baseline levels. At the same time, liver and kidney function indicators (AST, creatinine, urea nitrogen, etc.) reflecting organ dysfunction also showed the same trend. This coexistence of multiple critical markers strongly suggests that this type of patient may be trapped in a vicious cycle driven by “immune thrombosis”, in which the uncontrolled inflammatory response, diffuse microthrombosis, and the consequent organ ischemia combine to lead to the final adverse outcome. The platelet trajectory model constructed in this study just captures the overall impact of this vicious circle from the dynamic dimension - the persistent low-level platelet state is a comprehensive embodiment of the persistence and difficulty of reversing this pathological process.

We further performed detailed subgroup analyses based on the patient's baseline (age, sex, and comorbidities) and disease severity (SOFA, APACHE II, GCS score). The results showed that the association between platelet trajectories and mortality risk was particularly prominent in patients older than 75 years, females, APACHE II > 20 points, SOFA > 8 points, and GCS scores ≤ 12 points. In other subgroups, although the risk ratios of some groups were not statistically significant compared with the reference group, the risks were consistent from high to low, showing the trend of “sustained low level group> high level decline group > rebound rising group”. In addition, no significant interaction between the above factors and platelet trajectory grouping was detected. This result suggested that the prognostic value of platelet trajectories may be more clinically important in vulnerable patient groups with more severe disease and limited physiological reserves. And together, they confirmed the robustness of the association between platelet trajectories and prognosis. The consistent risk trend across subgroups and the absence of significant interactions suggested that the association might be a universal pattern that is not easily modified by the effects of common confounders such as age, gender, underlying disease, and initial disease severity. This provided a basis for generalizing the model to a wider population of sepsis. Our mediation analysis revealed that the degree of organ dysfunction represented by the SOFA score was an important mediating pathway connecting initial platelets to the risk of death. This finding was consistent with previous studies that platelets cause organ dysfunction during sepsis through a variety of mechanisms, including activation and aggregation to form blood clots, interaction with immune cells to form “danger alliances”, induction of NET formation, release of inflammatory mediators and procoagulants, and circulatory disorders (4). These mechanisms work together to eventually triggered microvascular dysfunction and organ damage. From a clinical point of view, the effect of prolonged coagulation time on prognosis might not be simply linear, but showed a “multiplication” effect, that was, when APTT breaks through a certain critical point, the risk of death associated with its small increase will increase sharply. This finding strongly suggested that the use of log (APTT) might be more risk-differentiated than the original value in future prognostic models or clinical decisions.

This study classified the dynamics of platelets in sepsis patients using a population trajectory model. Although the use of growth mixture models to analyze longitudinal data is not novel, the core value of this study lies in moving beyond methodological repetition, providing new clinical evidence and deeper mechanistic insights. First, we independently validated the strong association between platelet dynamic phenotypes and outcomes in a specific critical care cohort, offering new support for the generalizability of this theory across different populations. More importantly, the unique trajectory identified by us, termed “rebound rise,” provides a dynamic phenotype with a positive prognostic significance, enriching the understanding of the pathophysiological evolution in such patients. Furthermore, this study did not stop at classification itself but, through systematic mediation analysis, revealed the specific mediating role of organ dysfunction (SOFA score) and nonlinear coagulation activation (log (APTT)) in linking initial platelet dynamics to mortality risk. This finding advances the association from mere phenotype correlation to potential causal mechanisms, offering a new perspective on the complex role of coagulation parameters in sepsis. Therefore, the core innovation of this study does not lie in the first application of the method but in systematically addressing the progressive questions of “what are the characteristics of the phenotypes” and “what are the potential underlying mechanisms” using this method, accumulating richer evidence for the heterogeneity and precise prognostic judgment of sepsis.

In addition to circulating biomarkers, non-invasive imaging techniques are increasingly recognized as valuable tools for functional assessment and prognostic stratification in critical illness. Advanced MRI-based approaches, including hyperpolarized gas imaging and functional ventilation–perfusion assessment, enable real-time visualization of regional lung function and tissue perfusion without radiation exposure. These modalities provide spatial and physiological information that cannot be captured by blood-based indicators alone. Recent studies have demonstrated the feasibility of hyperpolarized gas MRI for quantitative evaluation of pulmonary ventilation and gas exchange, highlighting its potential role in risk prediction and disease monitoring (30, 31). Therefore, integrating imaging-derived functional metrics with laboratory trajectories may further enhance personalized prognosis assessment in critically ill patients.

Several limitations of this study should be acknowledged. First, study design and generalizability: This single-center observational study had a limited sample size—especially in the “rebound up” group—limiting generalizability. Selection and information biases are inherent to this design and cannot be fully eliminated. Although rigorous statistical modeling adjusted for many known confounders, residual confounding remains possible. Key clinical factors—including pathogen type, antimicrobial timing/selection, and vasoactive agent dosing—were not fully captured and may affect both platelet dynamics and outcomes. In this study, we did not perform statistical analyses on the pathogenic agents (such as bacteria and viruses) in sepsis patients. As is widely recognized, viral pathogens can modulate the host immune response via mechanisms like immune dysregulation, endothelial damage, and coagulation abnormalities, which might influence the severity and prognosis of the disease. In the future, stratified analysis of pathogens should be conducted to further investigate the differences in the prognostic impacts between bacterial sepsis and viral sepsis. Second, data quality and trajectory estimation. Modeling population trajectories involves subjective decisions—especially in selecting the optimal number of trajectory groups, which requires balancing statistical fit with clinical interpretability. All lab variables (e.g., platelet counts) came from routine clinical tests and thus carry inherent measurement error. Sampling times followed clinical practice—not standardized research protocols—potentially adding noise to trajectory identification. Third, limitations in analytical and causal inference. Mediation analysis offers insights for mechanistic exploration, but observational data cannot establish definitive causality—such studies are better suited for generating hypotheses than testing them. While variable selection and log (APTT) transformation are justified biologically and statistically, this mediation model remains just one plausible approach. Furthermore, “persistently low” platelet trajectory was strongly linked to higher admission SOFA scores and mortality. However, because severe organ dysfunction can itself cause ongoing platelet consumption and suppressed production, this trajectory may reflect underlying disease severity rather than independently drive poor outcomes. Its independent prognostic value—beyond initial disease severity—requires further validation in rigorously designed studies. Fourth, GBTM identifies temporal patterns—not mechanism-based subphenotypes—summarizing common trends statistically, not as discrete biological entities. Relying solely on platelet count is oversimplified: it ignores platelet function, production kinetics (e.g., reticulated platelets), and interactions with endothelial or coagulation pathways. Thus, the resulting phenotypes are descriptive only—useful for hypothesis generation, not comprehensive biological characterization. Although we applied established criteria to select clinically interpretable trajectories, results remain model - dependent. Future work should use higher-resolution longitudinal sampling, sensitivity analyses under alternative distributional assumptions, more flexible frameworks (e.g., growth mixture models), and multi-omics or systems-level approaches to validate trajectory stability and uncover underlying biology. Finally, trajectory construction used only platelet counts from the first three days after admission. While this window captures early disease dynamics, it may miss later platelet changes under treatment. Therefore, the identified trajectories represent an early acute-phase phenotype and require validation—using longer follow-up and denser sampling—for predictive stability and long-term applicability.

However, these limitations have not detracted from the distinct contribution of this study. Our study incorporated repeated longitudinal measurements and applied trajectory modeling to characterize temporal phenotypes, thereby identifying clinically meaningful patterns of platelet dynamics rather than relying solely on single baseline values. This longitudinal phenotyping framework may better reflect underlying pathophysiological heterogeneity and provide additional insights into disease mechanisms and prognostic pathways. By integrating dynamic trajectories with clinical outcomes and mechanistic exploration, we established a systematic analytical framework that offers a complementary perspective for understanding sepsis heterogeneity. The identified mediating effect and nonlinear relationship of log (APTT) have delineated the direction for future research. Although external validation in larger multi-center studies is warranted, this dynamic phenotypic classification strategy is anticipated to offer novel tools for more precise prognostic assessment and patient stratification in clinical trials.

## 5 Conclusion

This study successfully identified three clinical phenotypes with marked differences in prognosis by modeling and analyzing the early admission dynamic trajectories of platelet counts in sepsis patients. The study verified that the platelet dynamic trajectories based on continuous measurements can effectively identify high - risk populations. Among them, the “persistently low - level” phenotype is strongly associated with the highest degree of organ dysfunction and death risk. The mediating analysis further disclosed the possible mediating role of organ dysfunction and non - linear changes in coagulation parameters. Despite the constraints of this single - center retrospective study, it provides a novel dynamic perspective for the risk stratification of sepsis. The constructed trajectory model is expected to become a practical instrument for clinical prognostic assessment and has also indicated an important direction for subsequent mechanism research.

## Data Availability

The raw data supporting the conclusions of this article will be made available by the authors, without undue reservation.
