# Supplementary material for: Group-based trajectory modeling of platelet dynamics in sepsis: from phenotypic identification to the exploration of prognostic mediators
Source: Front Med (Lausanne). 2026 Feb 25;13:1761545. doi: 10.3389/fmed.2026.1761545 (PMC12975996; doi:10.3389/fmed.2026.1761545)
Supplement: Supplementary file 1 [file Data_Sheet_1.docx]

| Table S1. Goodness-of-fit indices for the group-based trajectory models of platelet counts in patients with sepsis | | | | | |
| --- | --- | --- | --- | --- | --- |
|  | **1-Group** | **2-Group** | **3-Group** | **4-Group** | **5-Group** |
| AIC | 11803.54 | 11750.72 | 11712.7 | 11698.86 | 11700.37 |
| BIC | 11828.98 | 11794.34 | 11774.5 | 11778.82 | 11798.51 |
| Relative entropy | NA | 0.67 | 0.80 | 0.87 | 0.78 |
| Average Posterior Probability (AvePP) | NA | 0.85/0.88 | 0.90/0.86/0.83 | 0.90/0.87/0.87/0.82 | 0.89/0.58/0.87/0.84/0.85 |
| Percent of patients with posterior probability <70% (per trajectory group) | 0 | 88.17/81.91 | 93.53/78.57/73.91 | 94.9/87.5/77.78/72.88 | 90.96/22.73/81.25/80/77.27 |
| Percent of sample size (per trajectory group) | 100 | 66.43/33.57 | 71.79/20/8.21 | 70/5.71/3.21/21.07 | 59.29/7.86/5.71/3.57/23.57 |
| AIC: the Akaike Information Criterion, BIC:the Bayesian Information Criterion (BIC) | | | | | |

| Table S2. Comparative Analysis of Baseline and Clinical Features Across Platelet Trajectory Subgroups | | | | | | |
| --- | --- | --- | --- | --- | --- | --- |
|  |  | Overall | Group1 | Group2 | Group3 | *p* |
| Sample Size |  | 280 | 201 | 56 | 23 |  |
| Baseline characteristics |  |  |  |  |  |  |
| Age (years) |  | 75 (65-82.25) | 76 (65-83) | 73.5 (60.5-80) | 73.52±10.04 | 0.28 |
| GCS (points) |  | 12 (5-15) | 12 (5-15) | 13.5 (5.75-15) | 12 (6-15) | 0.82 |
| SOFA (points) |  | 8 (5-12) | 9 (6-13) | 6 (4-9) | 8 (4.5-12) | <0.01 |
| APACHE II (points) |  | 20 (16-27) | 20 (16-27) | 21.5 (14.75-28.25) | 20.43±7.17 | 0.56 |
| gender (%) | Female | 96 (34.29) | 69 (34.33) | 16 (28.57) | 11 (47.83) | 0.26 |
|  | Male | 184 (65.71) | 132 (65.67) | 40 (71.43) | 12 (52.17) |  |
| hypertension (%) | Yes | 142 (50.71) | 100 (49.75) | 31 (55.36) | 11 (47.83) | 0.73 |
|  | No | 138 (49.29) | 101 (50.25) | 25 (44.64) | 12 (52.17) |  |
| diabetes (%) | Yes | 89 (31.79) | 64 (31.84) | 18 (32.14) | 7 (30.43) | 0.99 |
|  | No | 191 (68.21) | 137 (68.16) | 38 (67.86) | 16 (69.57) |  |
| Cardiac Insufficiency (%) | Yes | 77 (27.50) | 56 (27.86) | 14 (25.00) | 7 (30.43) | 0.87 |
|  | No | 203 (72.50) | 145 (72.14) | 42 (75.00) | 16 (69.57) |  |
| Serum indicators |  |  |  |  |  |  |
| Neutrophil (10^9^/L) |  | 10.28 (7.02-15.63) | 9.7 (6.8-15.7) | 11.58±6.57 | 12.8 (10.49-16.55) | 0.21 |
| Lymphocyte (10^9^/L) |  | 0.64 (0.4-0.95) | 0.63 (0.4-0.9) | 0.6 (0.5-0.95) | 0.94±0.6 | 0.18 |
| Monocyte (10^9^/L) |  | 0.5 (0.28-0.75) | 0.4 (0.21-0.72) | 0.5 (0.35-0.77) | 0.5 (0.38-1) | 0.19 |
| PLT (10^9^/L) |  | 155.5 (112.75-214.25) | 132.22±49.07 | 248.25±60.16 | 286.52±68.27 | <0.01 |
| Base PLT (10^9^/L) |  | 192 (142-245) | 166.17±52.7 | 261.46±70.02 | 316.87±51.96 | <0.01 |
| 24h PLT (10^9^/L) |  | 134.5 (96-193) | 114.61±43.43 | 250.52±46.13 | 211.3±71.5 | <0.01 |
| 48h PLT (10^9^/L) |  | 126 (88.75-178.25) | 106.93±46.28 | 229.32±66.72 | 212.26±97.65 | <0.01 |
| 72h PLT (10^9^/L) |  | 133.5 (83.75-192.75) | 114.75±54.6 | 199.2±82.69 | 260.74±95.62 | <0.01 |
| Min PLT (10^9^/L) |  | 92.5 (60.75-141) | 83.46±43.36 | 153.46±73.32 | 186±76.7 | <0.01 |
| MPV (fL) |  | 11.24±1.55 | 11.6±1.54 | 10.42±1.04 | 10.11±1.35 | <0.01 |
| PT (s) |  | 13.25 (12.1-14.8) | 13.5 (12.2-15.1) | 12.79±1.36 | 13.5±2.07 | <0.01 |
| APTT (s) |  | 40.5 (32.2-46.32) | 43.2 (36.5-50.1) | 32.85 (29.05-40.52) | 29.65±5.05 | <0.01 |
| FIB (g/L) |  | 4.44 (3.55-5.6) | 4.44 (3.68-5.69) | 4.6±1.69 | 4.1±1.73 | 0.13 |
| D-Dimer (μg/mL) |  | 3.58 (2.18-8.28) | 4.22 (2.44-8.59) | 2.41 (1.52-6.03) | 3.2 (2.63-6.23) | 0.01 |
| Organ function-related indicators |  |  |  |  |  |  |
| TBil (μmol/L) |  | 11.45 (7.8-19.12) | 13 (8.4-22.9) | 11.28±5.44 | 7.2 (4.65-12.1) | <0.01 |
| HDL (mmol/L) |  | 1.06 (0.74-1.33) | 1.05±0.43 | 1.14±0.47 | 0.93±0.32 | 0.14 |
| LDL (mmol/L) |  | 1.46 (1.09-1.86) | 1.45 (1.03-1.85) | 1.57 (1.24-2.22) | 1.58 (1.05-1.9) | 0.01 |
| Oxygenationindex (mmHg) |  | 232.88 (169.7-315) | 260 (170.9-326) | 220.75 (173.25-278.12) | 223.75±103.13 | 0.23 |
| ALB (g/L) |  | 29.5 (26.2-32.92) | 29.7±5.56 | 29.6 (27.3-33.73) | 27.75 (25.23-29.6) | 0.10 |
| ALT (U/L) |  | 25 (14-53.08) | 26 (15-55) | 20 (12-36.25) | 36 (12.5-62) | 0.10 |
| AST (U/L) |  | 37 (23-75.75) | 40 (25-92) | 26 (18.75-43.25) | 43 (23.75-88) | <0.01 |
| Cr (μmol/L) |  | 112.5 (67.75-185) | 124 (74.51-205.2) | 89 (54.75-177.36) | 86 (51.35-121.5) | 0.01 |
| BUN (mmol/L) |  | 10.44 (7-16.98) | 11 (7.3-17.51) | 8.85 (6.4-15.03) | 11.26±7.84 | 0.12 |
| Inflammatory markers |  |  |  |  |  |  |
| IL-6 (pg/mL) |  | 115.54 (22.15-264.82) | 119.69 (25.51-262.11) | 104.61 (18.52-302.58) | 87.14 (11.37-235.2) | 0.62 |
| PCT (mg/L) |  | 2.23 (0.47-8.7) | 2.85 (0.82-14.07) | 1.06 (0.21-3.27) | 2.06 (0.42-7.32) | <0.01 |
| WBC (10^9^/L) |  | 11.3 (7.96-17.2) | 11.3 (7.98-17.2) | 12.59±6.6 | 15.23±8.05 | 0.27 |
| CRP (mg/L) |  | 90.66 (30.12-162.09) | 91.34 (33.9-167.2) | 69.31 (20.81-127.58) | 108.93 (82.89-163.58) | 0.08 |
| Lac (mmol/L) |  | 2.8 (2-4.2) | 3 (2.1-4.7) | 2.2 (1.6-3.12) | 2.5 (2.15-3.25) | <0.01 |
| Serum electrolyte levels |  |  |  |  |  |  |
| Na (mmol/L) |  | 139.7 (135.7-142.5) | 139.99 (135.7-142.6) | 138.3 (135.75-142.06) | 139.57±7.25 | 0.73 |
| CI (mmol/L) |  | 101.2 (97.9-105.76) | 101.2 (98.1-106.3) | 100.7 (96.92-104.25) | 102.86±6.41 | 0.41 |
| Ca (mmol/L) |  | 1.95 (1.84-2.09) | 1.93 (1.83-2.08) | 1.99 (1.89-2.11) | 1.93±0.2 | 0.15 |
| K (mmol/L) |  | 3.9 (3.55-4.35) | 3.88 (3.54-4.31) | 4.11±0.67 | 3.82±0.48 | 0.09 |

| Table S3. Baseline Characteristics of Patients by 28-day Survival Status | | | | |  |
| --- | --- | --- | --- | --- | --- |
|  |  | Survival Group | Non-Survival Group | *p* | |
| Sample Size |  | 98 | 182 |  | |
| Baseline characteristics |  |  |  |  | |
| Age (year) |  | 75.5 (67.25-82) | 75 (63-83) | 0.63 | |
| GCS (points) |  | 12 (6-15) | 12 (5-15) | 0.99 | |
| SOFA (points) |  | 7 (4-11) | 9 (6-13) | <0.01 | |
| APACHE II (points) |  | 20 (15.25-26.75) | 21 (16-27) | 0.24 | |
| gender (%) | Female | 31 (31.63) | 65 (35.71) | 0.58 | |
|  | Male | 67 (68.37) | 117 (64.29) |  | |
| hypertension (%) | Yes | 53 (54.08) | 89 (48.90) |  | |
|  | No | 45 (45.92) | 93 (51.10) | 0.48 | |
| diabetes (%) | Yes | 29 (29.59) | 60 (32.97) |  | |
|  | No | 69 (70.41) | 122 (67.03) | 0.66 | |
| Cardiac Insufficiency (%) | Yes | 30 (30.61) | 47 (25.82) |  | |
|  | No | 68 (69.39) | 135 (74.18) | 0.47 | |
| Serum indicators |  |  |  |  | |
| Neutrophil (10^9^/L) |  | 9.73 (6.45-14.65) | 10.55 (7.35-16.67) | 0.22 | |
| Lymphocyte (10^9^/L) |  | 0.7 (0.5-1.15) | 0.6 (0.38-0.9) | 0.02 | |
| Monocyte (10^9^/L) |  | 0.5 (0.32-0.8) | 0.4 (0.2-0.72) | 0.05 | |
| PLT (10^9^/L) |  | 188 (147.5-256) | 134 (98.25-189.75) | <0.01 | |
| basePLT (10^9^/L) |  | 212 (174-286.25) | 170.5 (129.25-210) | <0.01 | |
| 24hPLT (10^9^/L) |  | 160.5 (131-236) | 116 (84.5-171) | <0.01 | |
| 48hPLT (10^9^/L) |  | 148.5 (110.25-207.75) | 106.5 (78.25-160.5) | <0.01 | |
| 72hPLT (10^9^/L) |  | 160 (115.75-209) | 112.5 (70-181) | <0.01 | |
| minPLT (10^9^/L) |  | 121 (83.25-157.75) | 82.5 (52-128.25) | <0.01 | |
| MPV (fL) |  | 10.83±1.46 | 11.46±1.55 | <0.01 | |
| PT (s) |  | 13.32±1.94 | 13.4 (12.2-15.07) | 0.08 | |
| APTT (s) |  | 33.6 (28.15-42.42) | 43 (36.6-48.7) | <0.01 | |
| FIB (g/L) |  | 3.72±1.06 | 5 (4.03-6.23) | <0.01 | |
| D-Dimer (μg/mL) |  | 3.08 (1.8-6.79) | 4.04 (2.41-9.12) | 0.02 | |
| Organ function-related indicators |  |  |  |  | |
| TBil (μmol/L) |  | 10 (7.32-16.32) | 12.6 (8.4-21.27) | 0.01 | |
| HDL (mmol/L) |  | 1.11±0.45 | 1.04±0.42 | 0.19 | |
| LDL (mmol/L) |  | 1.43 (1.09-1.74) | 1.48 (1.05-1.91) | 0.51 | |
| Oxygenationindex (mmHg) |  | 249.13±107.25 | 234.75 (169.44-311.88) | 0.97 | |
| ALB (g/L) |  | 28.9 (26-32.2) | 29.9 (26.52-33.32) | 0.17 | |
| ALT (U/L) |  | 21 (12-45.75) | 27.5 (15-54) | 0.15 | |
| AST (U/L) |  | 33.75 (22-52.75) | 39 (23.7-96.5) | 0.04 | |
| Cr (μmol/L) |  | 88.9 (55.25-135.75) | 131.01 (75.5-216.38) | <0.01 | |
| BUN (mmol/L) |  | 8.9 (6.4-14.5) | 11.5 (7.55-17.28) | 0.02 | |
| Inflammatory markers |  |  |  |  | |
| IL-6 (pg/mL) |  | 68.81 (15.65-340.96) | 135.54 (31.56-241.09) | 0.19 | |
| PCT (mg/L) |  | 1.62 (0.28-5.86) | 2.83 (0.85-11.93) | <0.01 | |
| WBC (10^9^/L) |  | 11.3 (7.25-16.38) | 11.3 (8.31-17.63) | 0.29 | |
| CRP (mg/L) |  | 92.03 (23.34-146.24) | 90.42 (37.31-167.43) | 0.44 | |
| Lac (mmol/L) |  | 2.6 (1.9-3.27) | 3 (2.02-4.8) | 0.01 | |
| Serum electrolyte levels |  |  |  |  | |
| Na (mmol/L) |  | 139.13 (134.9-142.45) | 139.94 (135.9-142.48) | 0.24 | |
| CI (mmol/L) |  | 101.32±7.97 | 101.05 (98.12-105.71) | 0.76 | |
| Ca (mmol/L) |  | 1.94 (1.83-2.09) | 1.96 (1.85-2.1) | 0.72 | |
| K (mmol/L) |  | 4.1 (3.7-4.52) | 3.89±0.64 | <0.01 | |
| Outcome-related indicators |  |  |  |  | |
| ICU Length of Stay (days) |  | 18 (9-30) | 7 (4-11) | <0.01 | |
| Hospital Length of Stay (days) |  | 26 (16-39) | 10.5 (7-19) | <0.01 | |
| 28-day survival time (days) |  | 28 (28-28) | 7 (4-12) | <0.01 | |
| platelet recovery (%) | Yes | 88 (89.80) | 124 (68.13) | <0.01 | |
|  | No | 10 (10.20) | 58 (31.87) |  | |
| AKI (%) | Yes | 32 (32.65) | 78 (42.86) | 0.12 | |
|  | No | 66 (67.35) | 104 (57.14) |  | |
| Shock (%) | Yes | 33 (33.67) | 68 (37.36) | 0.63 | |
|  | No | 65 (66.33) | 114 (62.64) |  | |

| Figure S1. Nonlinearity test between Continuous variables and 28 - day survival status | |
| --- | --- |
| 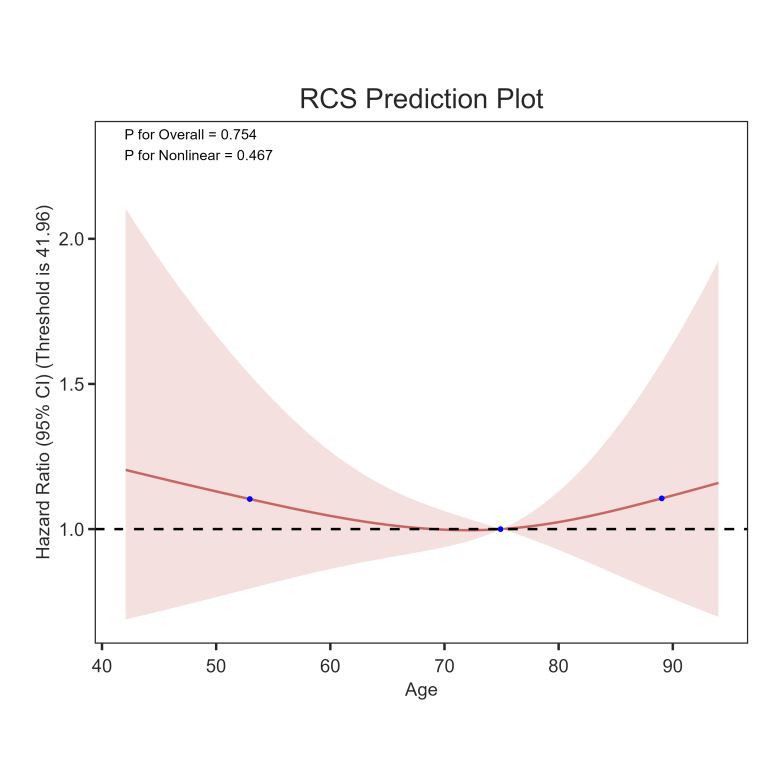 | 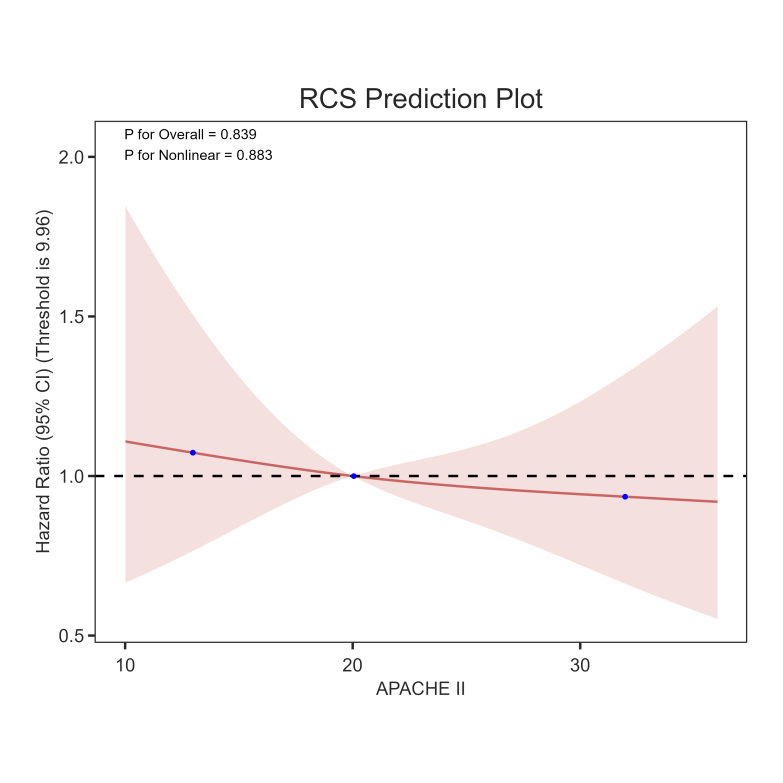 |
| 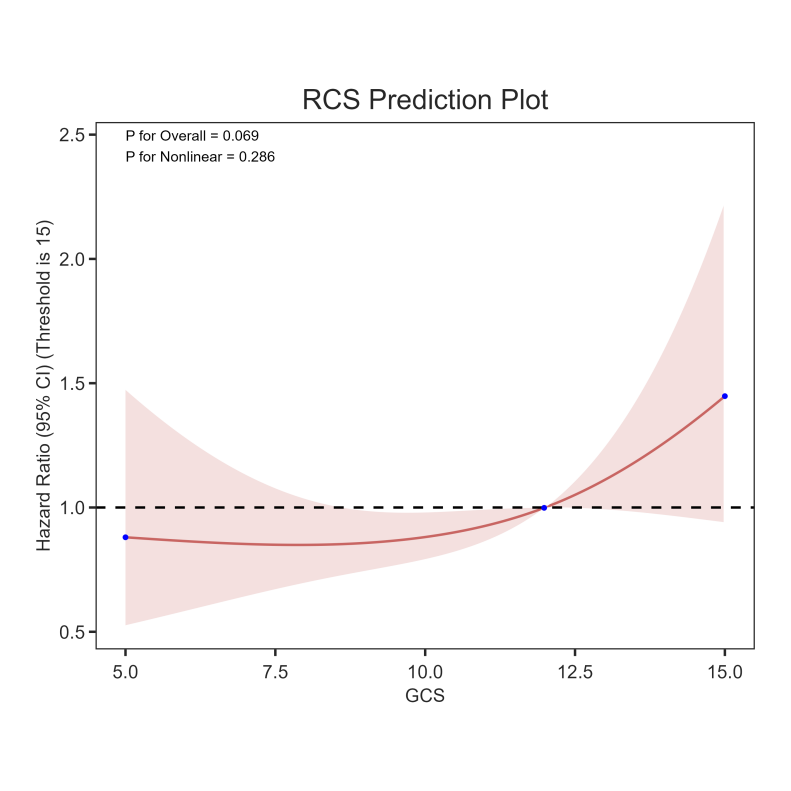 | 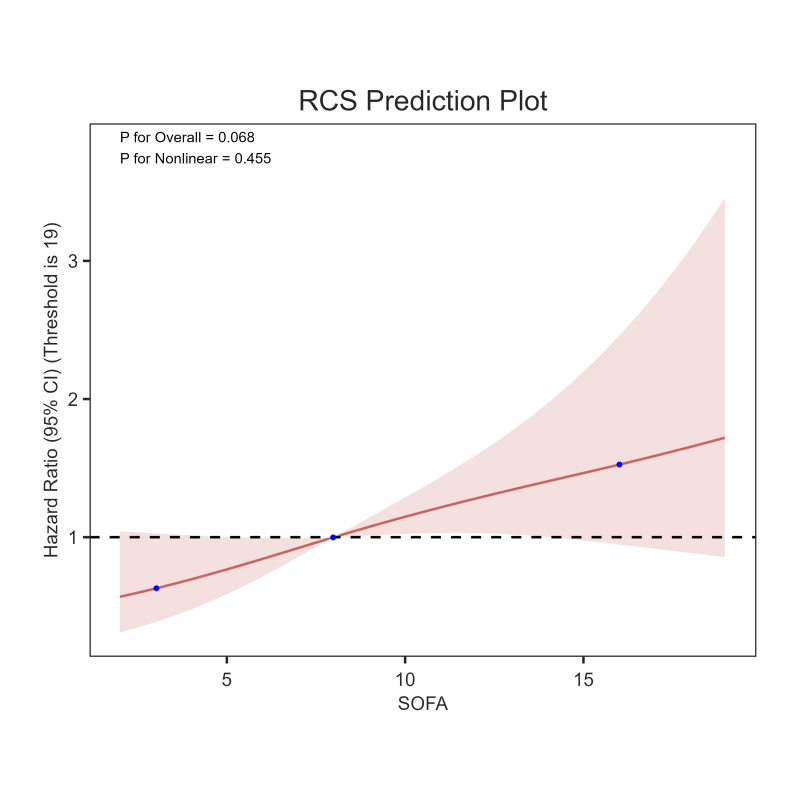 |

| Table S4. Nonlinearity test between Continuous variables and 28 - day survival status | | | |
| --- | --- | --- | --- |
| Row Name | Chi_Square | Df | P value |
| Age | 0.56 | 2 | 0.75 |
| Nonlinear | 0.53 | 1 | 0.47 |
| APACHE II | 0.35 | 2 | 0.84 |
| Nonlinear | 0.02 | 1 | 0.88 |
| SOFA | 5.37 | 2 | 0.068 |
| Nonlinear | 0.56 | 1 | 0.46 |
| GCS | 5.35 | 2 | 0.07 |
| Nonlinear | 1.14 | 1 | 0.29 |
| Neutrophil | 1.09 | 1 | 0.295 |
| Lymphocyte | 1.23 | 1 | 0.27 |
| Monocyte | 0.59 | 1 | 0.44 |
| minPLT | 6.11 | 1 | 0.01 |
| MPV | 0.56 | 1 | 0.46 |
| FIB | 36.13 | 1 | <0.001 |
| D-Dimer | 2.69 | 1 | 0.10 |
| Gender | 0.89 | 1 | 0.35 |
| hypertension | 1.35 | 1 | 0.25 |
| diabetes | 0.01 | 1 | 0.93 |
| cl | 2.92 | 1 | 0.09 |
| PCT | 0.08 | 1 | 0.78 |
| WBC | 0.08 | 1 | 0.78 |
| CRP | 3.50 | 1 | 0.06 |
| Lac | 2.27 | 1 | 0.13 |
| oxygenationindex | 0.81 | 1 | 0.37 |
| Sodium | 0.04 | 1 | 0.84 |
| Cl | 0.00 | 1 | 0.99 |
| ALB | 4.59 | 1 | 0.03 |
| Cr | 0.39 | 1 | 0.53 |
| Group | 15.84 | 2 | 0.00 |
| TOTAL.NONLINEAR | 2.56 | 4 | 0.63 |
| TOTAL | 88.56 | 30 | <0.001 |
